# Misidentification of *Burkholderia pseudomallei* as *Acinetobacter* species in northern Thailand

**DOI:** 10.1093/trstmh/try108

**Published:** 2018-10-05

**Authors:** Rachel C Greer, Tri Wangrangsimakul, Premjit Amornchai, Vanaporn Wuthiekanun, Achara Laongnualpanich, David A B Dance, Direk Limmathurotsakul

**Affiliations:** 1 Mahidol Oxford Tropical Medicine Research Unit, Faculty of Tropical Medicine, Mahidol University, Bangkok, Thailand; 2 Centre for Tropical Medicine and Global Health, Nuffield Department of Medicine, University of Oxford, Oxford, UK; 3 Chiang Rai Prachanukroh Hospital, Chiang Rai, Thailand; 4 Department of Tropical Hygiene, Faculty of Tropical Medicine, Mahidol University, Bangkok, Thailand; 5 Lao-Oxford-Mahosot Hospital-Wellcome Trust Research Unit, Microbiology Laboratory, Mahosot Hospital, Vientiane Capital, Lao People’s Democratic Republic; 6 Faculty of Infectious and Tropical Diseases, London School of Hygiene and Tropical Medicine, London, UK

**Keywords:** *Acinetobacter*, *Burkholderia pseudomallei*, laboratory quality assurance, melioidosis, misidentification

## Abstract

**Background:**

*Burkholderia pseudomallei* is the causative agent of melioidosis, a disease endemic throughout the tropics.

**Methods:**

A study of reported *Acinetobacter* spp. bacteraemia was performed at Chiang Rai provincial hospital from 2014 to 2015. Isolates were collected and tested for confirmation.

**Results:**

A total of 419 putative *Acinetobacter* spp. isolates from 412 patients were re-identified and 5/419 (1.2%) were identified as *B. pseudomallei*. Four of the five patients with melioidosis died. An estimated 88/419 (21%) isolates were correctly identified as *Acinetobacter* spp.

**Conclusions:**

Misidentification of *Acinetobacter* spp. as *B. pseudomallei* or other bacteria is not uncommon and programmes to address these shortfalls are urgently required.

## Introduction

*Burkholderia pseudomallei*, an environmental Gram-negative bacterium, is the causative agent of melioidosis.^1^ There are an estimated 165 000 human cases with 89 000 deaths annually worldwide.^2^ It is thought to be endemic in northern Thailand; however, only sporadic reports have emerged to date.^2^ Transmission occurs through percutaneous inoculation, inhalation or aspiration.^1^ Risk factors include diabetes mellitus, alcoholism, chronic lung disease and chronic kidney disease.^3^

Clinical misdiagnosis of melioidosis regularly occurs due to its protean manifestations, ranging from mild fever to fatal septic shock.^1,3^ Diagnosis is typically made by culture of clinical specimens and identification of *B. pseudomallei* using conventional microbiological techniques including Gram stain, oxidase test, biochemical tests and antibiotic susceptibility tests.^4,5^ Additionally, latex agglutination, identification kits (e.g. API20NE), matrix-assisted light desorption/ionization time-of-flight mass spectrometry (MALDI-TOF) and molecular tests can provide further confirmation.^5^ However, laboratory misidentification can occur due to lack of awareness, inadequate quality assurance or limited resources. Treatment involves an intensive phase with intravenous (IV) ceftazidime or meropenem for 10–14 d followed by an eradication phase of 3–6 months with oral co-trimoxazole or co-amoxiclav.^1^ Here we report five culture-confirmed cases whose *B. pseudomallei* had been misidentified as *Acinetobacter* spp.

## Materials and methods

A study of reported *Acinetobacter* spp. bacteraemia was conducted at Chiang Rai provincial hospital, in northern Thailand, from December 2014 to December 2015. Clinical blood culture isolates originally identified as *Acinetobacter* spp. by the local hospital laboratory using conventional microbiological techniques were prospectively collected. These isolates were subcultured and subsequently re-identified using standard biochemical tests for confirmation. Additional testing with a *B. pseudomallei*–specific latex agglutination test, VITEK 2 (bioMérieux, Durham, NC, USA) or MALDI-TOF were performed where necessary. Following laboratory confirmation of the identification of the isolates, clinical data were collected retrospectively and recorded on approved case record forms.

## Results and discussion

A total of 419 blood culture isolates reported as *Acinetobacter* spp. were obtained from 412 patients, of which 88/419 isolates (21%) were found to be oxidase positive (*Acinetobacter* spp. should be oxidase negative). Five of these (5.7%) were identified as *B. pseudomallei* based on the typical colonial appearance on Ashdown agar, positive latex agglutination test and VITEK 2 and MALDI-TOF profiles. The remaining oxidase-positive isolates comprised a variety of environmental bacteria, including *Achromobacter denitrificans* (22/88 [25.0%]), *Ralstonia mannitolilytica* (16/88 [18.2%]) and *Ochrobactrum anthropi* (9/88 [10.2%]). Of the 331/419 oxidase-negative isolates (79%), a sample of 60 isolates was tested using MALDI-TOF, confirming the identification as *Acinetobacter* spp. in 16/60 (26.7%) and *Stenotrophomonas maltophilia* in 43/60 (71.7%), with 1 isolate unidentified. If the sample set was representative, an estimated 88/419 (21.0%) isolates were initially correctly identified as *Acinetobacter* spp.

Clinical summaries of the five misidentified melioidosis cases are presented in Table 1 and details of the blood culture results for each patient in Table 2. Admission blood cultures were reported within 22–40 h of collection as *Acinetobacter* spp., while antibiotic susceptibility results were reported 16–24 h later. All had underlying disease associated with an increased risk of melioidosis. Four cases had severe disease, were elderly, developed multi-organ failure and died (patients 1, 2, 4 and 5). The initial empirical treatment in these patients included IV ceftriaxone or IV piperacillin/tazobactam with additional antibiotics in some cases (e.g. amikacin, roxithromycin, azithromycin, doxycycline and vancomycin). Only two of the four severe cases received melioidosis-appropriate antibiotics—IV meropenem in both cases—with delays of 1 and 4 d from admission (patients 2 and 5, respectively). The other two severe cases died within 48 h of admission (patients 1 and 4). The patient who survived was younger, had mild disease, had previously been diagnosed with genitourinary tract melioidosis and was already on appropriate treatment (IV ceftazidime), which was continued despite the misleading admission blood culture result (patient 4).

**Table 1.** Clinical summary, radiological findings and outcomes of five blood culture–confirmed melioidosis cases in Chiang Rai, Thailand (observations and SOFA scores on admission are shown)

Patient	Age (y)	Sex	Risk factors	Temperature (°C)	HR (/min)	BP (mmHg)	RR (/min)	SOFA score	Presenting symptoms	Examination findings	Radiological findings	Working diagnoses	Outcome
1	65	M	Smoker, chronic obstructive pulmonary disease, lung abscess	37.5	122	100/60	40	8	Fever, cough, dyspnoea, abdominal pain	Reduced breath sounds on the left; right upper quadrant abdominal pain	CXR: left lung abscess; US: hepatic parenchymal disease	Chronic lung abscess with acute pneumonia, septic shock, multi-organ failure	Died within 24 h of admission
2	62	M	Smoker, diabetes mellitus (new diagnosis)	35.0	66	69/45	20	9	Fever, abdominal pain, vomiting	Marked right upper quadrant tenderness with hepatomegaly	CXR: right upper zone infiltration	Septic shock, acute respiratory distress syndrome, multi-organ failure	Died on day 6 of admission
3	35	M	Smoker, alcoholism, cirrhosis	37.8	114	125/73	18	2	Fever	Hepatomegaly	Not done	Ongoing melioidosis, urinary tract infection	Recovered
4	64	M	Diabetes mellitus	36.6	125	130/68	Intubated and ventilated	14	Fever, cough, dyspnoea	Right lung crepitation	CXR: bilateral lung infiltrates	Septic shock, diabetic ketoacidosis, pneumonia, multi-organ failure	Died within 48 h of admission
5	62	M	Chronic kidney disease	38.5	107	109/73	Intubated and ventilated	13	Fever, cough, dyspnoea	Widespread wheezing and crepitation	CXR: right lung infiltration	Sepsis, pneumonia, multi-organ failure	Died on day 6 of admission

BP: blood pressure; CXR: chest X-ray; HR: heart rate; M: male; RR: respiration rate; SOFA: sequential organ failure assessment; US: ultrasound.

**Table 2.** Details of the blood culture results reported for the five misidentified melioidosis cases from Chiang Rai, Thailand

Patient	Collection time (day 0=admission)	Report time (hours after collection)	Results	Antibiotic susceptibility (S = Sensitive, I = Intermediate sensitivity, R = Resistant)
1	Day 0	37 h	*Acinetobacter lwoffii*	S—ceftazidime, imipenem, cefoperazone/sulbactam R—amikacin, gentamicin, co-trimoxazole
2	Day 0	28 h	Mixed growth: *A. lwoffii*, *Staphylococcus epidermidis*	S—ceftazidime, imipenem, piperacillin-tazobactam, cefoperazone/sulbactam, ciprofloxacin, ertapenem, meropenem, cefotaxime, co-trimoxazole R—amikacin, gentamicin NB—susceptibility results for *Acinetobacter lwoffii* only
	Day 2	99 h	*Burkholderia pseudomallei*	S—ceftazidime, imipenem, cefoperazone/sulbactam R—amikacin, gentamicin, co-trimoxazole
3	Day 0	40 h	*Acinetobacter baumannii*	S—ceftazidime, doripenem, imipenem, piperacillin-tazobactam, cefoperazone/sulbactam, ertapenem, meropenem, cefotaxime R—amikacin, gentamicin, co-trimoxazole
4	Day 0	22 h	*A. baumannii*	S—ceftazidime, doripenem, imipenem, piperacillin-tazobactam, cefoperazone/sulbactam, ertapenem, meropenem, cefotaxime R—amikacin, gentamicin, co-trimoxazole
5	Day 0	25 h	*A. lwoffii*	S—ceftazidime, doripenem, imipenem, piperacillin-tazobactam, cefoperazone/sulbactam, ertapenem, meropenemI—cefotaxime R—amikacin, gentamicin, co-trimoxazole

NB: antibiotic susceptibility results were generally reported 16–24 h after the blood culture flagged positive.

In this report we confirm the presence of melioidosis in northern Thailand and demonstrate that laboratory misidentification of *Acinetobacter* spp. as *B. pseudomallei* or other bacteria remains commonplace. The predicted mortality from melioidosis in Thailand is 37.5%, while 4/5 (80%) patients in this study died.^2^ This confirms the high mortality associated with melioidosis, especially when compounded by misdiagnosis and delays in initiating effective treatment. However, earlier initiation of appropriate antibiotics alone, without timely and adequate intensive care support, may not improve the outcome in severe disease.^3^ Empirical ceftriaxone and piperacillin/tazobactam were used: ceftriaxone has moderate *in vitro* activity against *B. pseudomallei*, while susceptibility to piperacillin/tazobactam has also been shown.^6,7^ Despite these findings, ceftriaxone is less effective than ceftazidime and clinical experience with piperacillin/tazobactam is limited.^8^ Correct identification on admission blood culture might have improved the outcome in one patient where appropriate treatment was delayed for 4 d. Even in endemic areas, microbiology laboratories can struggle to identify *B. pseudomallei*. Training and introduction of a simple laboratory algorithm have been shown to be effective in diagnosing melioidosis in Vietnam.^4^ During the study period, nine other patients had melioidosis diagnosed from positive blood cultures, demonstrating that local laboratory staff are capable of identifying *B. pseudomallei*, albeit inconsistently. In addition, this study shows that data from routine laboratory-based surveillance systems, even in countries like Thailand with relatively well-developed health care facilities, should be treated with caution.

## Conclusions

Making the correct diagnosis is vital to patient outcome and fundamental to any effective surveillance and antimicrobial stewardship programmes. The diagnosis and management of melioidosis remain suboptimal in many endemic regions. The misidentification of *B. pseudomallei* and other bacteria demonstrated in this report highlights the need to prioritize and strengthen laboratory capacity and quality. Following discussions with local stakeholders, plans are under way to provide additional training, improve quality assurance and introduce consistent use of new diagnostics (e.g., latex agglutination test, API20NE and VITEK) within the microbiology laboratory. Efforts to increase the clinical awareness of melioidosis, including the importance of effective and timely treatment in at-risk patients, are also required.
